# Perfectionism, the Impostor Phenomenon, Self-Esteem, and Personality Traits among Russian College Students

**DOI:** 10.11621/pir.2023.0310

**Published:** 2023-09-30

**Authors:** Marina S. Sheveleva, Tatiana M. Permyakova, Dmitriy S. Kornienko

**Affiliations:** a Department of Foreign Languages, National Research University Higher School of Economics, Perm, Russia; b Institute for Social Sciences Russian Presidential Academy of National Economy & Public Administration, Moscow, Russia

**Keywords:** impostor phenomenon, perfectionism, the Big Five personality traits, mediation, self-esteem

## Abstract

**Background:**

Perfectionism and the Impostor Phenomenon (IP) have mainly been studied in American samples, as have the associations of Perfectionism and the Impostor Phenomenon with Self-Esteem and the Big Five personality traits. However, previous studies showed that results might depend on cultural background. There is a critical lack of such research in the Russian context which might limit generalization of the previous findings to a narrow range of cultures.

**Objective:**

In this study, the authors investigated how Perfectionism and the Impostor Phenomenon are related to the 5-factor model of personality, and examined the mediating role of Self-esteem between the dimensions of Perfectionism and the Impostor Phenomenon, using a Russian sample.

**Design:**

The study sample comprised 372 undergraduate students age 18–23 (M = 19.07, SD = 1.05). The Impostor Phenomenon, Personality Traits, and Self-Esteem were measured by relevant questionnaires.

**Results:**

The results indicated that Adaptive Perfectionism had a strong positive correlation with Extraversion, Conscientiousness, and Openness. Maladaptive Perfectionism had a strong relation to Conscientiousness and Neuroticism. Neuroticism demonstrated a strong positive correlation with impostor tendencies and was the main predictor. Self-esteem partially mediated the link between Maladaptive Perfectionism and the Impostor Phenomenon, intensifying negative feelings and Impostorism.

**Conclusion:**

These results generally replicated the pattern from previous studies of the relationship between Perfectionism, the Big Five personality traits, Self-esteem, and the Impostor Phenomenon. Thus, it could be possible to conclude that the studied relationships might be regarded as universal for the Russian students in terms of culture.

## Introduction

Perfectionism is considered to be a widespread phenomenon (Stoeber & Stoeber, 2009; Stricker, Buecker, Schneider, & Preckel, 2019), and is increasing as new generations as young people face more demands from society or their parents (Curran & Hill, 2019). The definition of this phenomenon is twofold. It is described as an excessive striving for excellence, combined with an overly critical attitude toward one’s results (Stoeber & Otto, 2006).

Perfectionism as a multidimensional construct has been studied since the 1990s (Smith et al., 2022). Three models of multidimensional Perfectionism have generated the vast majority of the research. The first model relies on the Frost Multidimensional Perfectionism Scale (FMPS) (Frost, Marten, Lahart & Rosenblate, 1990) and includes six dimensions: concerns over mistakes, doubts about actions, personal standards, parental criticism, parental expectations, and organization. The second model, proposed by Hewitt and Flett (1991), is built around the Hewitt and Flett Multidimensional Perfectionism Scale (HFMPS). It includes three types of Perfectionism: self-oriented Perfectionism, other-oriented Perfectionism, and socially-prescribed Perfectionism. The third model was proposed by Slaney, Rice, Mobley, Trippi, and Ashby in 2001. The scale employed is the Almost Perfect Scale (APS) with its variations: Almost Perfect Scale-Revised (APS-R) and Short Almost Perfect Scale (SAPS). This scale includes two dimensions: Standards and Discrepancy. Respondents who have high scores on the Standards subscale but low scores on the Discrepancy sub-scale are referred to as “Adaptive Perfectionists.” Respondents who have high scores on both subscales are referred to as “Maladaptive Perfectionists.”

The APS measure is found to be a reliable instrument for assessing Perfectionism multidimensionally (Cokley et al., 2015) and has demonstrated good psychometric properties in adaptations to other languages. It has been used to study Perfectionism in different countries: Holland (Van Yperen & Hagedoorn, 2008); Japan (Nakano, 2009); Korea (Park, 2009); Turkey (Öngen, 2009); and Russia (Wang, Permyakova, & Sheveleva, 2016). In this study, we followed the third model of Perfectionism, due to its proven track record for research in a range of countries (Rice, Loscalzo, Giannini & Rice, 2018).

In order to streamline the research on Perfectionism, Stoeber and Otto (2006) operationalized two dimensions of Perfectionism in the three models described above. The first dimension is called “perfectionistic concerns,” or “Maladaptive Perfectionism,” and includes the following subscales: concerns over mistakes; doubts about actions (Frost et al., 1990); socially prescribed perfectionism (Hewitt et al., 1991); and discrepancy (Slaney et al., 2001). The second dimension is called “perfectionistic strivings,” or “Positive Perfectionism,” and includes personal standards, parental expectations, and organization (Frost et al., 1990), self-oriented Perfectionism (Hewitt et al., 1991), and high standards (Slaney et al., 2001).

The construct of Impostorism has received significant attention in the literature over the past decades. A systematic literature review has revealed over 1,200 studies of the Impostor Phenomenon with 80% being published in this millennium (Mak, Kleitman, & Abbott, 2019). The Impostor Phenomenon, or Impostorism, can be defined as the inclination to think that one has reached a professional success because of luck, continuous effort, or some kind of mistake – but not due to one’s intellectual abilities (Clance, 1985; Pannhausen, Klug & Rohrmann, 2020). Employees with Impostorism fear that they will be exposed as “frauds” and are often prone to anxiety, low self-confidence, depression, and frustration (Clance & Imes, 1978; Stone-Sabali, Bernard, Mills, & Osborn, 2023).

The studies of the Impostor Phenomenon could be grouped into three fields: organizational and environmental settings typical for this phenomenon (Bernard & Neblett, 2018; Chakraverty, 2020; Neureiter & Traut-Mattausch, 2017; Parkman, 2016; Sharma, 2018); its links with other personality dispositions (Ferrari & Thompson, 2006; McGregor, Gee, & Posey, 2008; Schubert & Bowker, 2019; Yaffe, 2020); and psychometric properties of the Impostor Scale (Chrisman, Pieper, Clance, Holland & Glickauf-Hughes, 1995; French, Ullrich-French, & Follman, 2008; Simon & Choi, 2018), and its adaptations to other languages (Brauer & Wolf, 2016; Chae, Piedmont, Estadt & Wickset, 1995).

### Perfectionism and the Big Five personality traits

The number of studies examining Perfectionism and the Big Five personality traits is quite extensive, and represents 25 years of research. The interest of researchers in this topic is explained by the need to place Perfectionism and its dimensions into a broader personality framework. The widely-used personality model includes Neuroticism, Extraversion, Openness, Agreeableness, and Conscientiousness (John, & Srivastava, 1999). Neuroticism exhibits an emotional mood and excitability. Extraversion is expressed by characteristics of sociality and mobility. Openness relates to imagination, acceptance of new ideas, and mental curiosity. Agreeableness indicates trustworthiness and altruism. Conscientiousness reflects self-discipline and a tendency to be responsible. (McCrae & Costa (2008). Two recent meta-analytic articles (Smith et al., 2019; Stricker et al, 2019) report over 75 independent studies of Perfectionism and the five-factor model of personality. The key finding was that regardless of the chosen Perfectionism model, in most studies perfectionistic concerns are correlated with neuroticism, low agreeableness, and low extraversion, while perfectionistic strivings are correlated with conscientiousness. At the same time, many inconsistencies in research were noted, largely resulting from the chosen Perfectionism scale and the sample size (Smith et al., 2019).

Due to the studies of Smith et al. (2019) and Stricker et al. (2019), the populations that were underrepresented in areas research could be clearly identified. Firstly, 85% out of 77 reported studies were based on American, Canadian, Australian, and British samples, while only the remaining 15% of studies included speakers of languages other than English. Respondents in these separate studies were from Turkey, Belgium, Germany, and China. Secondly, 54 studies followed Hewitt and Flett’s instrumental understanding of Perfectionism and employed the Hewitt and Flett Multidimensional Perfectionism Scale (HFMPS), and 29 studies applied Frost’s Perfectionism model, employing the Frost Multidimensional Perfectionism Scale (FMPS). Only 15 studies followed a different Perfectionism model proposed by Slaney et al. (2001) and employed a well-established Almost Perfect Scale, and its forms, namely Almost Perfect Scale-Revised (APS-R) or Short Almost Perfect Scale (SAPS). Many studies employed several scales in one article, which inflated the results, producing a total greater than 77.

If we analyze the studies with different forms of the Almost Perfect Scale, two out of those 15 studies included psychiatric and medical patients samples (Békés et al., 2015; Chang, 2009), while 13 studies employed non-clinical samples, and will be of particular relevance to our research. In order to provide reliable findings in cross-sectional studies, the sample size is required to be larger than 250 participants (Schönbrodt & Perugini, 2013). The sample size in the 13 studies under analysis varied from 84 to 1,465 respondents. However, only in four studies was the sample size larger than 250 participants (Clark, Lelchook, & Taylor, 2010; Dunkley, Blankstein, & Berg, 2012; Rice, Richardson, & Tueller, 2014; Ulu, & Tezer, 2010). It is also worth noting that in terms of participants’ origin, the population sample was again narrowed. In the 13 studies reviewed, the sampled populations were mainly of American, Canadian, and Australian origin. Only three studies included respondents from non-English-language backgrounds (Ozbilir, 2011; Ozbilir, Day, & Catano, 2015; Ulu & Tezer, 2010).

In Turkey, Ulu & Tezer (2010) used a sample of 604 undergraduate students. The results showed that high Standards were positively correlated with Extraversion, Conscientiousness, and Openness, while Discrepancy was negatively correlated with Extraversion and positively with Neuroticism. These results followed the same pattern as the results of Rice et al. (2014) with one exception. In the latter study, Discrepancy also had a strong negative correlation with Conscientiousness. Clark et al. (2010) conducted a similar study on the sample of 323 working university students from one American university. Their findings showed that high Standards were significantly positively related to Extraversion, Agreeableness, Conscientiousness, and Openness, while Discrepancy was significantly negatively related to Agreeableness, Conscientiousness, and Neuroticism. Dunkley et al. (2012) do not report the inter-correlations between the study variables. The summary of variables’ intercorrelations in the research overview is provided in *Table 1.*

**Table 1:** Intercorrelations between Discrepancy, Standards and the Five Factor Model Dimensions

Study			Intercorrelations with Big 5				
Authors and year	Sample size and origin	Subscales	Extra-version	Agreeable	Conscientiousness	Neuroticism	Openness to experience
Ulu & Tezer, 2010	604 university students, Turkey	Standards	.19*	.05	.41*	.20	.32*
		Discrepancy	–.16*	–.05	–.03	.40*	–.09
Clark, Lelchook, & Taylor, 2010	323 university students, USA	Standards	.22**	.47**	.49**	–.10	.52**
		Discrepancy	–.08	–.18**	–.24**	–.40**	–.09
Rice, Richardson, & Tueller, 2014	340 university students, USA	Standards	.23**	.09	.46**	–.05	0,37**
		Discrepancy	–.32**	.11	–.22**	.59**	.09

Regression analysis demonstrated that Conscientiousness, Openness, and Extraversion were the main predictors for Adaptive Perfectionism, while Maladaptive Perfectionism was predicted by Neuroticism to a large extent (Ulu & Tezer, 2010).

The goal of the present research is to build on these four studies by providing a different cultural context and addressing the inconsistencies present in the previous works.

### Impostorism and the Big Five personality traits

A number of articles have explored the relationship of the Impostor Phenomenon (IP) to other personality constructs (e.g., Fried-Buchalter, 1992; King & Cooley, 1995). Within this research context, the IP is connected to a range of traits including the Big Five Personality Model (Watson, 2012; Vergauwe, Wille, Feys, De Fruyt, & Anseel, 2015). However, the number of studies exploring the relatedness of the IP to the Five-Factor Model of personality is limited. To the best of our knowledge, there have been only four studies based on a range of samples in terms of their origin: Korean (Chae et al., 1995); American (Ross, Stewart, Mugge, & Fultz, 2001, and Bernard, Dollinger & Ramaniah, 2002); and Belgian (Vergauwe et al., 2015).

A strong positive relationship between the IP scales and Neuroticism and a strong negative relationship between the IP scales and Conscientiousness were observed in all four studies (Chae et al., 1995; Ross et al., 2001; Bernard et al., 2002; Vergauwe et al., 2015).

However, findings based on other traits of the Big Five taxonomy were inconsistent. Extraversion had significant, but low, negative correlations with the Clance Impostor Phenomenon Scale (CIPS) in some studies (Chae et al., 1995, Ross et al., 2001, Vergauwe et al., 2015), but not in others (Bernard et al., 2002). Meaningful low correlation with Agreeableness was shown only by Chae et al., 1995.

The inconsistencies mentioned above could be explained by a number of factors, including the sample size (from 129 in Ross et al., 2001, to 654 in Chae et al., 1995); sample type – consisting of working adults (Chae et al., 1995; Vergauwe et al., 2015) as opposed to college students (Bernard et al., 2002; Ross et al., 2001); scales used to measure the IP and the Big Five; and the sample origin.

Given the inconsistencies described above, and the possibility that Impostorism might depend on cultural background (Chae et al., 1995), the replication of the results with a Russian sample could contribute to our existing knowledge.

### The link between Perfectionism and the Impostor Phenomenon

Many studies mention the connection between Perfectionism and the Impostor Phenomenon, as both share a number of symptoms such as setting unattainable high standards, fear of failure, self-criticism, absence of satisfaction with good performance, procrastination, and low Self-esteem (Hill et al., 2004; Cokley et al., 2015; Lane, 2015; Pannhausen, Klug, & Rohrmann, 2020). However, there is scant research in this area. Thompson, Foreman, and Martin (2000) showed a strong link between impostor fears and perfectionistic concerns over mistakes, as well as the role of Perfectionism in predicting and maintaining the Impostor Phenomenon. In more recent studies, it has been shown that Maladaptive Perfectionism or perfectionistic concerns (and not Adaptive Perfectionism or perfectionistic strivings) predict the development of the Impostor Phenomenon (Dudau, 2014; Pannhausen et al., 2020). These findings were deepened by Wang, Sheveleva, & Permyakova (2019), who showed that the Impostor Phenomenon is the key link between perfectionistic discrepancy and negative mental health outcomes such as depression and anxiety.

Despite the well-studied association between Impostorism and Perfectionism, the mechanism behind this relationship remains unknown. Self-esteem has been found to mediate the relationship between Perfectionism and other characteristics. For instance, Self-esteem mediates the relationship between Adaptive Perfectionism and work-family conflict (Deuling & Burns, 2017). Prior research on the relationship between the Impostor Phenomenon and Self-esteem has yielded rather varied results. Schubert and Bowker (2017) demonstrated the crucial role of Self-esteem and Self-esteem instability in the Impostor Phenomenon. Some studies have discovered the mediating role of Self-esteem in relation to Impostorism and racial identity (Lige et al., 2017), impostor sentiments, and parenting styles (Yaffe, 2020).

Only one study addresses the nature of the relationship between Perfectionism and the Impostor Phenomenon (Cokley et al., 2018). The authors hypothesized that Self-esteem might be the link between the Impostor Phenomenon and Perfectionism. They demonstrated that Self-esteem was a partial mediator for the link between Perfectionism and the Impostor Phenomenon. The authors stated that this study needs to be replicated due to its limitations such as the sample origin (American in this case). These findings and limitations motivated us to reproduce the study in a different cultural setting.

Thus, the preliminary, inconsistent results (Stricker et al., 2019), limited samples (Smith et al., 2019), predominantly English-speaking respondents from the United States, the United Kingdom, and Canada (Methikalam, Wang, Slaney, & Yeung, 2015), and the existence of cross-cultural differences (Chae et al., 1995), substantiate the need for study of the following research questions:

RQ1: How do the Discrepancy and Standards subscales relate to the Big Five personality model in a Russian sample?

RQ2: How does the Impostor Phenomenon relate to the Big Five personality model in a Russian sample?

RQ3: Does Self-esteem mediate the relationships between Perfectionism and Impostorism in a Russian sample?

## Methods

### Participants

The participants were 372 undergraduate students (277 female — 74.5%) between 18 and 23 years of age (M = 19.07, SD = 1.05) from Russian universities. The respondents majored in a range of subjects: Chemistry, Economics, Engineering, IT, Math, Management, Law, and Psychology. There were non-significant differences in age (t = –.695, ns.) between men (M = 19.02, SD = .09) and women (M = 19.09, SD = .07).

### Procedure

The subjects were tested in a group session during regular class hours. They received a brief introductory talk about the study’s aims, completed online questionnaires, and provided their demographic details. They were instructed to take as long as needed to complete the questionnaires, and it took participants an average of 20 minutes to complete them. The questionnaires were filled out in the presence of the researcher. Upon completion, the participants were debriefed and thanked. Their participation was voluntary, and no compensation was paid. The respondents received course credit as an incentive to participate in research.

The questionnaires were filled out in Russian, as all of them were either developed in Russian (*Short Portrait Big Five Questionnaire* (Egorova & Parshikova, 2016) or adapted to the Russian language in previous studies (please see Sheveleva et al., 2021; Wang et al., 2016; Zolotareva, 2020, for reference). The study’s procedures complied with the ethical code for research of the institutions from which the participants were recruited.

### Materials

*The Clance Impostor Phenomenon Scale* (CIPS; Clance, 1985; Sheveleva et al., 2021*)* was used to assess Impostorism, a fear of being evaluated and failing to reproduce achievements, and the tendency to underestimate oneself. Items were anchored on a 1–5 Likert-type scale (1 = strongly disagree; 5 = strongly agree). The CIPS has strong reliability and validity (French et al., 2008; Mak et al., 2019). In this sample, Cronbach’s alpha was .89.

*The Rosenberg Self-esteem Scale* (RSES; Rosenberg, 1965; Zolotareva, 2020*)* is a unidimensional instrument elaborated from a phenomenological conception of Self-esteem that captures subjects’ global perception of their worth through a 10-item scale, rated on a 4-point Likert-type scale, ranging from 1 (strongly disagree) to 4 (strongly agree). Rosenberg (1965) reported Cronbach alphas from .85 to 0.88 for the samples of college students. Cronbach’s alpha for this study is .88.

*The Short Portrait Big Five Questionnaire* (BF-10; Egorova & Parshikova, 2016) is a 10-item domain-level personality scale designed to assess the Big-Five personality dimensions: Agreeableness, Conscientiousness, Openness, Extraversion, and Emotional Stability. Each item presents a description of a person with whom the respondents compare themselves using the 6-point Likert scale from 1 (this person is completely different from me) to 6 (this person is very much like me). The average internal consistency for all traits is .58. The average Cronbach’s alpha for this study is .51, which corresponds with other studies of the Big Five (*e.g.,* Romero, Villar, Gómez-Fraguela & López-Romero, 2012)

*The Short Almost Perfect Scale* (SAPS) is a brief, established measure of the Almost Perfect Scale-Revised (Slaney et al., 2001; Wang et al., 2016). The SAPS consists of two subscales: Standards and Discrepancy. The Standards subscale measures the level of perfectionistic striving by assessing one’s setting of high expectations. The Discrepancy subscale measures the level of perfectionistic concerns by assessing each participant’s tendency to perceive a gap between their standards and performance. Respondents were asked to rate each item on a seven-point Likert scale where 1 was “strongly disagree” and 7 was “strongly agree.” The Cronbach alphas ranged from .85 to .87 for Standards and .84 to .87 for Discrepancy (Rice et al., 2014). In the present study, the Cronbach alphas of Standards and Discrepancy scores were .82 and .79, respectively.

*Data analysis.* Pearson’s correlation and mediation analyses were carried out. The mediation analysis was conducted with PROCESS and the plugin for SPSS, based on the bootstrapping technique developed by Preacher and Hayes (2004). Bootstrapping is not based on a normal distribution; that is why a total of 5000 bootstrap samples were used to obtain 95% CIs (confidence interval) and test the significance of the indirect effect. The significance of the indirect effect was indicated if the 95% CI did not include zero.

## Results

Descriptive statistics including means, standard deviations, and correlations between all scales are presented in *Table 2.*

**Table 2:** Descriptive statistics and correlations of variables.

Variable	M	SD	1	2	3	4	5	6	7	8	9
1. Impostor Phenomenon	49.36	10.88									
2. Standards	21.22	4.69	–.031								
3. Dscrepancy	16.48	5.96	.528**	.164**							
4. Self-esteem	29.78	5.66	–.680**	.237**	-.540**						
5. Extraversion	8.02	2.61	–.252**	.221**	–.106*	.308**					
6. Agreeableness	8.97	2.07	–.152**	–.053	–.199**	.200**	.075				
7. Conscien- tiousness	8.75	2.35	–.165**	.192**	–.162**	.207**	–.031	.248**			
8. Neuroticism	6.55	2.32	.468**	.035	.311**	–.474**	.002	–.283**	–.148**		
9. Openness	8.72	2.03	–.373**	.371**	–.172**	.475**	.552**	.085	.045	–.207**	

Note. * — p<.05; ** — p<.001.

### Research question 1. Perfectionism and the Big Five personality traits

The Pearsons’ correlations were conducted to examine the relations between personality traits and Perfectionism variables. Standards positively correlated with Conscientiousness (r = .192; p<.01), Openness (r = .371; p<.01), Extraversion (r = .221; p<.01), and Discrepancy negatively associated with Agreeableness (r = –.199; p<.01), Conscientiousness (r = –.162; p<.01), Openness (r = –.172; p<.01), Extraversion (r = –.106; p<.01). Moreover, Discrepancy positively correlated with Neuroticism (r = .311; p<.01).

The multiple regression results showed that three Big Five traits accounted for a significant amount of the variance in high Standards (R2 = .18, F(5, 392) = 17.25, p<.001). Agreeableness (β = –.100; p<.05), Conscientiousness (β = .220; p<.001), Openness (β = .351; p<.001) revealed as a significant predictors for high Standards. Concerning the Big five predictors for Discrepancy Conscientiousness (β = –.12, p<.01), and Neuroticism (β = .237, p<.001) had a significant impact with 14% of the total amount of variance (R2 = .14, F(5, 392) = 12.26, p<.001).

### Research question 2. Impostorism and the Big Five personality traits

With regard to the mean Impostor tendencies shown in *Table 2*, a one-way ANOVA indicated no significant sex differences in mean Impostor tendencies F(1,370) = 8.16, p<.01. There was no association between the Impostor Phenomenon and age.

To examine the relation between Impostorism and personality traits, we calculated Pearson correlation coefficients. The intercorrelations of the variables are given in *Table 2.*

At the level of the zero-order correlations, it was found that Impostorism positively correlated with Neuroticism (r = .486; p<.01) and negatively with the other traits: Agreeableness (r = –.152; p<.01), Conscientiousness (r = –.165; p<.01), Openness (r = –.373; p<.01), Extraversion (r = –.252; p<.01).

Next, we conducted the multiple regression where the Big Five traits were entered as predictors for the Impostor, R2 = .32, F(5, 392) = 37.1 4, p<.001. When controlling for shared variance among the Big Five traits, Extraversion (β = –.13, p<.01), Conscientiousness (β = –.11, p<.01), Neuroticism (β = .40, p<.001), and Openness (β = –.22, p<.001) were still associated with impostor tendencies.

### Research question 3. Perfectionism and the Impostor Phenomenon

The correlations between the Impostor Phenomenon and Self-esteem showed a significant negative relationship (r = –.680; p<.01). Discrepancy was negatively correlated with Self-esteem (r = –.540, p<.01), and positively correlated with Impostorism (r = .528, p<.01). Standards were positively associated with Self-esteem (r = .237, p<.01) and insignificantly correlated with the IP.

### Mediation analyses

**Figure 1.**
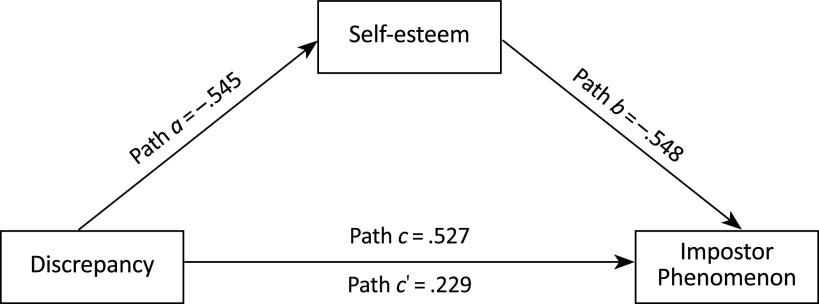
Mediation model of the indirect effect of discrepancy on the Impostor Phenomenon through Self-esteem

Following Cokley’s procedures (2018), two different mediation analyses with Self-esteem as a mediator between Perfectionism (Discrepancy and Standards) and Self-esteem were performed. The result of regression of Discrepancy on Impostorism was significant (b = .527, SE = .019, p<.001), and the subsequent regression of Discrepancy on Self-esteem was also significant (b = –.545, SE = .016, p<.001). Next, while controlling for Discrepancy, the regression of Self-esteem on Impostorism was found significant (b = –.548, SE = .016, p<.001). After controlling for Self-esteem, Discrepancy continued to be a significant predictor of Impostorism (b = .229, SE = .019, p<.001). Discrepancy and Self-esteem explained 48.8% of the variance of Impostorism (*Figure 1*). Therefore, we concluded that Self-esteem was a partial mediator between Discrepancy and Impostorism.

**Figure 2.**
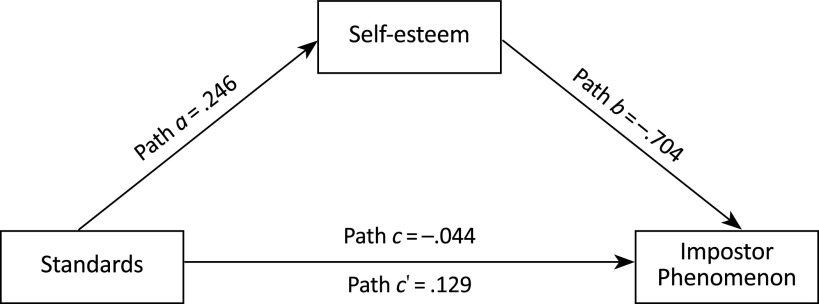
Mediation model of the indirect effect of standards on the Impostor Phenomenon through Self-esteem

The analytical procedure was repeated to test the direct effect of Standards and the indirect effect of Self-esteem on Impostorism. The regression of Standards on Impostorism was insignificant (b = –.044, SE = .028, p<.383); the regression of Standards on Self-esteem was significant (b = .246, SE = .024, p<.001). While controlling for Standards, the regression of Self-esteem on Impostorism was also significant (b = –.704, SE=.043, p<.001). Next, while controlling for Self-esteem, Standards appear to be the significant predictor for Impostorism (b = .129, SE = .021, p<.001). Standards and Self-esteem accounted for 46.7% of the variance of Impostorism ( *Figure 2*). The indirect effect of Standards on Impostorism was significant ( …. ) *b* = –.173, SE = .038, CI = –.246, –.098 ( …. )

### Assessment of mediation

The indirect effect of Discrepancy on Impostorism was significant ( …. ) *b* = .298, SE = .029, CI = .237, .355 ( …. )

## Discussion

The aim of this research was to examine the relationships between Perfectionism, the Impostor Phenomenon, and the Big Five personality traits with a Russian sample, as well as the mediating role of Self-esteem between Perfectionism and the Impostor Phenomenon.

### Perfectionism and the Big Five personality traits

The results of the present study are in line with previous research (Ulu & Tezer, 2010; Rice et al., 2014). In this study, Standards had a strong positive correlation with Extraversion, Conscientiousness, and Openness. The Standards subscale followed a consistent correlational pattern with previous studies showing that more social-oriented, open-minded, responsible, and self-controlled people pose higher standards for themselves in terms of performance results. People with high Standards tend to do their best and achieve competence in what they are doing. This fact is rooted in their personality traits.

Discrepancy does not show consistent correlations as different results are reported in a range of studies. However, the most replicable link is with Neuroticism. Our results correspond with Ulu & Tezer (2010) and Rice et al. (2014) that Conscientiousness and Neuroticism have a strong relationship with Discrepancy. Concerning the role of Extraversion in Discrepancy at the correlation level, we found the same results as Ulu & Tezer (2010), but Extraversion did not appear in the predictors model in this study.

The reasons why the Standards subscale showed a more stable pattern than Discrepancy could be based on the cultural origin of the respondents and a range of scales used to measure the Five Factor Personality Model. Moreover, any person could set standards while the Discrepancy scale shows the relationship between Standards and performance. It stems from the fact that personality traits could hinder the performance and achievements.

Overall, our study, in line with previous research, demonstrated that Adaptive Perfectionism (measured by the Standards subscale) was mainly linked with positive personality traits such as Conscientiousness and Openness, while Maladaptive Perfectionism (measured by the Discrepancy subscale) was connected with Neuroticism to a larger extent.

### Impostorism and the Big Five personality traits

To identify people with the highest Impostorism scores with the CIPS, the cutoff values from previous studies were used (Holmes, Kertay, Adamson, Holland, & Clance, 1993; Chae et al., 1995). Using the values of 58 and 62 it was found that only 20% and 13% of our sample could be regarded as Impostors. Such percentages are half as much as in the Korean sample, and crucially smaller than the American sample (Clance, 1985; Harvey & Katz, 1985). Even though these differences need to be studied more precisely, we may postulate (according to Chae et al., 1995) that cultural differences may influence Impostorism.

Our findings of Impostor and Neuroticism relations support the previous studies (Ross et al. 2001; Bernard et al., 2002; Vergauwe et al., 2015). Neuroticism demonstrates a strong positive correlation with Impostor tendencies and acts as the main predictor. The results of the relationship between Impostor and Agreeableness, Conscientiousness, and Extraversion cohere with studies of Chae et al. (1995), as we also found low but significant associations between Impostor and the mentioned personality traits. Based on the previous studies and our results, we support the idea that Neuroticism is the primary personality trait in relationships with the Impostor Phenomenon, whereas the other traits play a complementary role in predicting impostor tendencies. We may speculate that more emotionally unstable individuals could demonstrate more impostor tendencies such as experiencing anxiety, low self-confidence, and proneness to psychological distress.

### Perfectionism and the Impostor Phenomenon

The results of this study replicate Cokley et al. (2018) both in terms of correlations, regressions, and mediation analysis. The Discrepancy subscale had significant, positive correlations with the Impostor phenomenon. The Standards subscale was non-significantly, negatively correlated with the Impostor Phenomenon. Despite the expected cultural dependency of the results, current research on the relationship between Impostor and Perfectionism aligns with the findings from European and American samples (Wang et al., 2019).

The mediating effect of Self-esteem was the highest for Discrepancy. As Self-esteem mediates the link between Maladaptive Perfectionism and the Impostor Phenomenon, it intensifies negative feelings and Impostorism. It means that Maladaptive Perfectionists who have low scores on Self-esteem are prone to Impostorism. They worry about the gap between their high goals and real-life results and negatively evaluate themselves. This results in experiencing intellectual fraudulence, being highly self-critical, and attributing success to external factors, as well as higher anxiety, depression, and other negative mental health outcomes.

This discussion leads to two conclusions. First, low Self-esteem is clearly seen as a factor intensifying Impostorism. Thus, low Self-esteem should be the point of intervention during counselling work with clients suffering from Maladaptive Perfectionism and Impostorism. Secondly, the link between Perfectionism, Self-esteem, and Impostorism could be regarded as universal in terms of culture.

## Conclusion

This study leads to a better understanding of the links between Perfectionism, Impostorism, and the Big Five as well as Perfectionism and Impostorism with each other. Extraversion, Conscientiousness, and Openness had positive correlations with Standards. Conscientiousness had negative and Neuroticism positive relations with Discrepancy. Neuroticism was the primary personality trait in relationship with the Impostor Phenomenon. Self-esteem mediated the link between Maladaptive Perfectionism and the Impostor Phenomenon.

Comparing the results of this study with previous studies, we can state that the strongest patterns were replicated. This result could lead to the conclusion that these relationships exist notwithstanding the cultural background of the respondents, and might be regarded as universal in terms of culture.

This study generally replicated the result pattern from previous studies of the relationship between Perfectionism, the Big Five personality traits, Self-esteem, and the Impostor Phenomenon.

Practical implications of this study could be connected with providing counselling support to students in higher educational establishments in Russia. As students high on Maladaptive Perfectionism and Impostorism tend to drop out more often, understanding the point of intervention while providing counselling sessions might yield better results.

## Limitations

This study has some limitations. First, the convenience sample limits the generalizability. There were more women than men in the sample. Second, only self-reported measures were used. Third, a cross-sectional design is a limitation in itself.

Several paths for further research could be suggested. First, there might be cross-cultural studies on IP and Perfectionism and their trait-relatedness, controlling for other sample characteristics and measurements. Second, perspective studies may try to find other mediators between Perfectionism and Impostorism. Third, qualitative studies could provide a better understanding of the psychological nature of Perfectionism and Impostorism.
